# Phosphoinositide regulation of inward rectifier potassium (Kir) channels

**DOI:** 10.3389/fphys.2013.00404

**Published:** 2014-01-08

**Authors:** Oliver Fürst, Benoit Mondou, Nazzareno D'Avanzo

**Affiliations:** Groupe d'étude des Protéines Membranaires (GÉPROM), Physiologie, Université de MontréalMontréal, QC, Canada

**Keywords:** phosphoinositides, inward rectifier potassium channels, lipid protein interactions, Kir channel, ion channel, ion channel gating, ligand-gated ion channels

## Abstract

Inward rectifier potassium (Kir) channels are integral membrane proteins charged with a key role in establishing the resting membrane potential of excitable cells through selective control of the permeation of K^+^ ions across cell membranes. In conjunction with secondary anionic phospholipids, members of this family are directly regulated by phosphoinositides (PIPs) in the absence of other proteins or downstream signaling pathways. Different Kir isoforms display distinct specificities for the activating PIPs but all eukaryotic Kir channels are activated by PI(4,5)P_2_. On the other hand, the bacterial KirBac1.1 channel is inhibited by PIPs. Recent crystal structures of eukaryotic Kir channels in apo and lipid bound forms reveal one specific binding site per subunit, formed at the interface of N- and C-terminal domains, just beyond the transmembrane segments and clearly involving some of the key residues previously identified as controlling PI(4,5)P_2_ sensitivity. Computational, biochemical, and biophysical approaches have attempted to address the energetic determinants of PIP binding and selectivity among Kir channel isoforms, as well as the conformational changes that trigger channel gating. Here we review our current understanding of the molecular determinants of PIP regulation of Kir channel activity, including in context with other lipid modulators, and provide further discussion on the key questions that remain to be answered.

Inward rectifier potassium (Kir) channels are a family integral membrane proteins that selectively control the permeation of K^+^ ions across cell membranes. The 15 members of this subfamily are charged with key roles in establishing the resting membrane potential of excitable cells, regulation of pacing in cardiomyocytes and neurons, regulation of pancreatic insulin secretion, and renal K^+^ transport (Rougier et al., 1968; Beeler and Reuter, 1970; Gahwiler and Brown, 1985; Frindt and Palmer, 1989; Takahashi, 1990; Wang et al., 1990; Inagaki et al., 1995). All members of this family share a basic topology with four sub-units combining to form a canonical pore-forming transmembrane domain that is selective for K^+^ ions, a small aliphatic helix on the N-terminus termed the “slide-helix” thought to interact rest at the bilayer interface, and a large cytoplasmic domain that extends the ion conduction pathway and provides docking sites for regulatory ions, proteins, and ligands (Ho et al., 1993; Kubo et al., 1993; Kuo et al., 2003; Nishida et al., 2007; Tao et al., 2009; Hansen et al., 2011; Whorton and MacKinnon, 2011, 2013; Bavro et al., 2012). It is suspected that interactions between the cytoplasmic domain and slide-helix may be necessary for the mechanical transduction of ligand binding to channel gating (Decher et al., 2007). In 1996, K_ATP_ (Kir6.x) channels were the first channels to be identified as phosphatidylinositol 4,5-bisphosphate [PI(4,5)P_2_ or PIP_2_] dependent (Hilgemann and Ball, 1996).

## Phosphoinositides (PIPs)

Phosphoinsotides (PIPs) are acidic phospholipids that contain a *myo*-inositol headgroup, which can be phosphorylated at the 3, 4 or 5 positions of the inositol ring in every combination. This gives rise to seven phosphoinositides [including the parent unphosphorylated phosphatidylinositol (PI)] which are present in low abundance in eukaryotic cellular membranes (typically 1–10% of total membranes depending on the cell type and pathophysiological status) (Wheeldon et al., 1965; Singh and Swartwout, 1972; Galloway et al., 1987; Post et al., 1995; Hamplova et al., 2004). PIPs are essential and found primarily in the cytoplasmic leaflet of eukaryotic membranes (Nikawa and Yamashita, 1982, 1997; Nikawa et al., 1987), however, in bacterial membranes PI and phosphorylated derivatives are rarely found. In fact, within prokaryotes PI appears to be confined to some actinomycetes, myxobacteria, *B. japonicum* and *Treponema* (Sohlenkamp et al., 2003).

Arguably the best characterized lipid modulator of ion channel activity, including Kir channels, is PI(4,5)P_2_ (or PIP_2_) (Suh and Hille, 2008). This phosphoinositide contains three phosphate groups, and is expected to carry a net charge near −4 at neutral pH, though this can vary between −3, −4 and −5 depending on the lipid environment, and specific interactions with the protein (McLaughlin et al., 2002). While cleavage of PIP_2_ by phospholipase C (PLC) into the second messangers inositol 1,4,5-triphosphate (IP_3_) and diacylglycerol (DAG) can affect some ion channels function through downstream signaling pathways, or recruiting soluble proteins to the plasma membranes, our discussion will be limited to the direct effects of PIP_2_ and other PIPs on Kir channel function.

The molecular basis for PIP-protein interactions can arise through a variety of mechanisms, primarily consisting of a combination of specific co-ordinated interactions and non-specific electrostatic interactions. On one extreme, myristoylated anlanine-rich C kinase substrate (MARCKS) is a 331-residue natively unfolded protein with a cluster of 13 basic residues termed the “basic effector domain” that confers a strong local positive electrostatic potential to the protein (Tapp et al., 2005). When not phosphorylated, MARCKS can bind the acidic headgroups of PIP_2_ through non-specific electrostatic interactions, sequestering them laterally across the membrane (Wang et al., 2002a). Specificity for interacting with PIP_2_ likely results from the multiple negative charges in the lipid headgroup and because PIP_2_ is generally the most abundant multi-phosphorylated lipid in the plasma membrane. On the other end of the spectrum, pleckstrin homology domains (PH domains) are found in numerous cytoplasmic proteins. Typically containing just over 100 residues, all PH domains known have a common structure consisting of two perpendicular anti-parallel beta sheets followed by a C-terminal amphipathic helix. This fold forms a pocket that contains several basic amino acids that are positioned and oriented in a manner that specifically enable the co-ordination of a PIP. Typically, several basic residues will co-ordinate the phosphates around the inositol ring, while other hydrogen bond interactions may also occur between protein and lipid via uncharged residues (Lemmon, 2003). By placing the basic residues in different positions within the binding pocket, different PIP specificities can arise through differing co-ordination patterns of the headgroup phosphates. Over the years, many experiments have been performed to determine where in this spectrum lay the of possible molecular mechanisms involved in PIP regulation of Kir channels.

## PIP regulation of kir channels

K_ATP_ channels (composed of Kir6.2 and SUR2A subunits) were the first channels whose activity was determined to be modulated by PIP_2_ (Hilgemann and Ball, 1996). Since then, electrophysiological experiments determined that all Kir channels are regulated by PIPs, albeit with each channel isoform differing in sensitivity to the specific ligand isoforms (Rohacs et al., 1999, 2003). For example, while Kir2.1 channels are selectively activated by PI(4,5)P_2_, with only ~10% of maximal activity by PI(3,4,5)P_3_ and little or no activation by the remaining PIPs (Rohacs et al., 1999, 2003; D'Avanzo et al., 2010a), Kir3.1/3.4 channels are maximally activated by PI(4,5)P_2_, and can be activated to ~80% of maximal activity by PI(3,4,5)P_3_ and ~20–30% maximal activity by PI(3,4)P_2_ (Rohacs et al., 1999, 2003). On the other hand, Kir6.2 channels are equally activated by PI(4,5)P_2_, PI(3,4)P_2_, PI(3,4,5)P_3_, and long chain CoA (Fan and Makielski, 1997; Shyng and Nichols, 1998; Rohacs et al., 2003). Neither of these channels are activated or inhibited by PI alone (Fan and Makielski, 1997; Baukrowitz et al., 1998; Rohacs et al., 1999, 2003; D'Avanzo et al., 2010a; Cheng et al., 2011). Until recently, these sensitivities were examined by electrophysiological experiments using cellular systems, which unfortunately could not definitively exclude the possibility of channel regulation via indirect methods such as through protein kinase A (PKA) or protein kinase C (PKC) pathways, or other proteins. However, the absolute dependence of the activities of full-length human Kir2.1 and Kir2.2 channels on PI(4,5)P_2_, independent of other proteins or signaling pathways, was verified using purified protein reconstituted into lipid bilayer systems of defined composition (D'Avanzo et al., 2010a; Cheng et al., 2011). This was later confirmed in Kir3.1-KirBac chimeric channels (Leal-Pinto et al., 2010) and mouse Kir3.2 channels (Whorton and MacKinnon, 2013). Interestingly, the bacterial Kir channel, KirBac1.1, is inhibited rather than activated by PIPs (Enkvetchakul et al., 2005; Cheng et al., 2009; D'Avanzo et al., 2010a; Cheng et al., 2011), with increasing inhibition as the number of phosphates on the ligand increases (Enkvetchakul et al., 2005). Together, these data suggest a more PH domain like model of PIP regulation in Kir channels, whereby charge alone is important but not sufficient. Rather, somewhat specific interactions are needed for binding and activation/inhibition of the channels.

PIP regulation of all Kir channels also appears to involve more than just the headgroup, since IP_3_ alone does not alter channel activity (Shyng and Nichols, 1998; Rohacs et al., 1999; Enkvetchakul et al., 2005). Thus, tethering of the headgroup to the membrane does appear to be important, however, the sensitivity to the lipid tail depends on the isoform. While Kir2.1 channel activity does not differ between PI(4,5)P_2_ acyl tails containing arachidonic-stearic (AA-St or 20:4–18:0), dipalmitoyl (diC16:0), or dioleic (di18:1) acids (Rohacs et al., 1999; D'Avanzo et al., 2013), Kir3.1/3.4 channels are 4 times more active in the presence of AA-St PIP_2_ compared to diC16:0 PIP_2_ (Rohacs et al., 1999).

The apparent affinity of Kir channels for PIP_2_ can be modified by other intracellular effectors. Phosphorylation of Kir1.1 channels by PKA decreases the sensitivity to inhibition by PIP_2_ antibodies, indicating an apparent increase in affinity for PIP_2_ (Liou et al., 1999). Gβγ subunits of G-proteins and intracellular Na^+^ alter PIP_2_ sensitivity in Kir3.x channels (Huang et al., 1998; Zhang et al., 1999; Jin et al., 2008; Inanobe et al., 2010), while intracellular ATP decreases the apparent affinity of Kir6 channels for PIP_2_ by reducing the channel's open probability (Baukrowitz et al., 1998; Shyng and Nichols, 1998; Enkvetchakul et al., 2000; Shyng et al., 2000; Wang et al., 2002b).

PIP_2_ was shown to increase the open probability of K_ATP_ channels comprised of Kir6.2 + SUR1 subunits, reducing the probability of ATP binding (Baukrowitz et al., 1998; Shyng and Nichols, 1998). Similar increases on the open probability have also been shown for other members of the Kir channel family (Fan and Makielski, 1997; Huang et al., 1998; Shyng and Nichols, 1998; Rohacs et al., 1999; Leung et al., 2000; Cheng et al., 2009; D'Avanzo et al., 2010a). For most Kir channels, 1 or 2 open times and 1 or 2 closed times can readily be observed in single channel recordings depending on the specific isoform and recording conditions. With few exceptions (Fan and Makielski, 1997; Rohacs et al., 1999; Lopes et al., 2002), PIP_2_ has generally been found to affect one or more of the closed times with no change in the open time(s) (Enkvetchakul et al., 2000; Jin et al., 2008; Xie et al., 2008; D'Avanzo et al., 2010a) suggesting a critical role for this lipid ligand in priming the channels for opening upon binding to a closed conformation. Several atomic resolution structures solved by x-ray crystallography (Nishida et al., 2007; Tao et al., 2009; Hansen et al., 2011; Whorton and MacKinnon, 2011, 2013) provide further support for this gating model.

## PIP regulation of kir channels in context of other membrane lipids

The sensitivity of Kir2.1 and Kir2.2 channels to activation by PIP_2_ was recently found to depend on the concentration of other anionic phospholipids in the membrane (Cheng et al., 2011). Using purified full-length channels reconstituted into liposomes of defined composition, human Kir2.1 and Kir2.2 channels were found to be nearly 100-fold more sensitive to PIP_2_ in the presence of 25% anionic phospholipids PG, PA, PS, PI, and DGS-NTA than in their absence. This effect was dependent on the concentration of the anionic phospholipids present in the membrane, and since these lipids could not activate Kir2.1 and Kir2.2 channels in the absence of PIP_2_, this secondary anionic phospholipid dependence appears to be synergistic with the PIP_2_ requirement rather than result from these anionic lipids acting as PIP_2_ surrogates. In contrast to the singular effect of physiological concentrations of PIP_2_ on the open probability of Kir2.1 channels (i.e., no change in unitary conductance) (D'Avanzo et al., 2010a), increasing POPG from 15 to 25% on a 1% PIP_2_ background not only increases open probability but also increases unitary conductance (Cheng et al., 2011). Computational experiments suggest a putative binding site for these anionic phospholipids at the end of the slide helix (D'Avanzo et al., 2013; Schmidt et al., 2013) and away from the binding pocket in which PIP_2_ was observed in the Kir2.2 and Kir3.2 channel crystal structures (Hansen et al., 2011; Whorton and MacKinnon, 2011; Schmidt et al., 2013). This secondary requirement for anionic phospholipids may act to further stabilize interactions between the slide helix and cytoplasmic domain necessary for channel gating (Decher et al., 2007), and additionally shield positively charged residues that may exert a significant effect on ion energetics inside the pore through long-range electrostatics (Robertson et al., 2008).

PIPs appear to regulate Kir channels by acting on both modes of lipid regulation (D'Avanzo et al., 2010a; Cheng et al., 2011). In liposomes composed of supra-physiological levels of PIP_2_, Kir2.1 is partially activated, and the effect of POPG is diminished. This is consistent with PIP_2_ at high levels being able to meet both lipid requirements and thus having dual regulatory roles in Kir2.1 channels. Other PIPs can have competing effects on Kir2.1 activity depending on the lipid background. On a 89% POPE and 1% PIP_2_ background, all other PIPs at 10% can activate Kir2.1 channels, but with variable efficacy. In this condition, PI is the most effective, followed by monophosphorylated PIPs, with the least effective being multi-phosphorylated PIPs. By contrast, when the secondary anionic phospholipid requirement is already satisfied (with for example the presence of 25% POPG in the membranes), all other PIPs but not PI inhibit Kir2.1 activity, with multi-phosphorylated PIPs being most effective. As with oleoyl CoA, PIPs are most likely inhibiting Kir2.1 activity by antagonizing the primary PIP_2_ requirement. A recent study has identified the secondary anionic phospholipid site to be established by two lysine residues (K64 and K219) adjacent to the PIP binding site. When bound, these lipids likely tether the cytoplasmic domain to the membrane, enabling the action of PIP_2_ (Lee et al., 2013).

Cholesterol is the major sterol component of all mammalian plasma membranes, playing critical roles in cell function and growth. Cholesterol has been shown to inhibit several Kir channels possibly through locking the channels in a prolonged closed state (Romanenko et al., 2004; D'Avanzo et al., 2011). It is conceivable that cholesterol may inhibit Kir channels by interfering with Kir—PIP_2_ interactions. However, cholesterol effects on Kir2.1 current density were not correlated to neomycin-induced current rundown and cholesterol sensitivities of Kir2.1 and Kir2.3 channels were unaffected by decreased PIP2 availability by PH-PLCδ 1 sequestration (Epshtein et al., 2009). Cholesterol inhibition was also not affected by altering the concentration of the secondary anionic phospholipid (D'Avanzo et al., 2011). These observations suggest cholesterol regulation of Kir channels is PIP_2_-independent.

## Structural determinants of PIP sensitivity and selectivity

**Figure 1:**
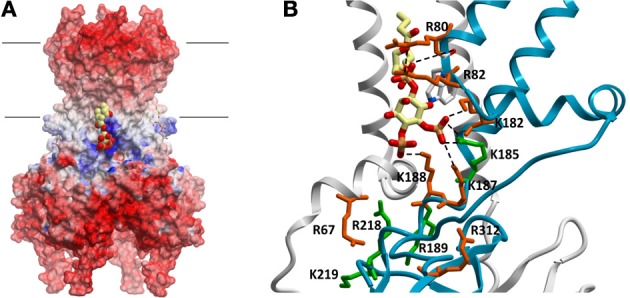
**(A)** Electrostatic potential map of chicken Kir2.2 channels (PDB:3SPI). Large regions of negative potentials are present at the extracellular and intracellular surfaces, which are important for ion and inhibitor binding. A band of positively charged residues lie just below the lipid bilayer which (i) form critical interactions necessary to establish a three dimensional pocket for PIP lipids, and (ii) in the case of some of these residues, help to co-ordinate the ligands in the pocket. PI(4,5)P_2_ molecules are shown in space-filling and stick representations in this binding pocket. **(B)** PI(4,5)P_2_ (and likely all other PIPs) is coordinated by residues at the interface of 2 subunits. Postively charged residues thought to contribute to PIP_2_ sensitivity are highlighted using Kir2.1 channel numbering. Despite their proximity and in some cases their involvement in co-ordinating the lipid, not all residues contribute to the energy of PIP_2_ binding. Residues in green contribute to the energetics of binding, while residues in red appear to primarily affect gating transitions [adapted from Hansen et al. (2011)].

The search for the molecular basis of PIP regulation of Kir channel activity has been a tribute to the wide range of experimental techniques available to study ion channels. Such experimental approaches include everything from electrophysiology, biochemical assays using truncated and full-length proteins, to computational docking and molecular dynamic simulations, and x-ray crystallography. To understand why Kir channels are activated by PI(4, 5)P_2_ it is necessary to identify the location and structure of the PIP binding site(s). Several studies have used mutagenesis combined with electrophysiology or biochemical assays on GST-fusion proteins of isolated Kir channel domains to identify molecular determinants of PI(4, 5)P_2_ regulation (Zhang et al., 1999; Cukras et al., 2002; Zeng et al., 2002; Rohacs et al., 2003; Pegan et al., 2005, 2006; Haider et al., 2007a; Nishida et al., 2007). Such studies have suggested that numerous positively charged residues in the N- and C-termini determine sensitivity of Kir channels to PI(4, 5)P_2_ activation (Huang et al., 1998; Rohacs et al., 1999; Zhang et al., 1999; Soom et al., 2001; Lopes et al., 2002; Zeng et al., 2002; Donaldson et al., 2003; Pegan et al., 2006; Xie et al., 2008). Using Kir2.1 channel numbering, these residues include H53, R67, R82, K182, K185, K187, K188, R189, R218, K219, K228, and R312. In most Kir channels, mutation of the equivalent residues in these positions to glutamine or alanine disrupt activation by PI(4, 5)P_2_. Electrostatic surface profiles of all Kir channels crystallized to date show a predictable band of positive charge just below the slide-helix and TM2 that can be attributed to most of these residues (Figure. 1A). Furthermore, atomic structures of Kir2.2 (Hansen et al., 2011) and Kir3.2 channels bound to PI(4, 5)P_2_ (Hansen et al., 2011; Whorton and MacKinnon, 2011, 2013) reveal one specific site, formed at the interface of N- and C-terminal domains, just beyond the transmembrane segments and clearly involving some but not all of these residues (Figure 1B). Thus it is clear that some of these residues affect PI(4, 5)P_2_ sensitivity primarily through affecting the gating of the channel rather than binding. However, these atomic structures cannot provide insight into the energetic contributions of the various residues to ligand binding. An attempt to address this was recently performed using direct binding approaches with full-length Kir2.1 channels (D'Avanzo et al., 2013). Despite the equivalent residues in Kir2.2 channels helping to co-ordinate the PI(4, 5)P_2_ ligand in the crystal structure, R82Q and K182Q, K187Q, K188Q mutations did not markedly affect the binding of PI(4, 5)P_2_ in Kir2.1 channels. H53Q, R67Q, and K228Q and R312Q mutations, which are all located outside of this binding pocket, but previously implicated in defining PIP_2_ sensitivity (Huang et al., 1998; Rohacs et al., 1999; Zhang et al., 1999; Soom et al., 2001; Lopes et al., 2002; Zeng et al., 2002; Donaldson et al., 2003; Pegan et al., 2006; Xie et al., 2008), also did not affect PI(4, 5)P_2_ binding. Thus, the role of these residues in regulating channel activity appears to be primarily in transducing the ligand binding event into the gating mechanism rather than affecting the energy of ligand binding. Instead, PI(4, 5)P_2_ binding in Kir2.1 channels appears to be controlled by K185, R189, R218 and K219 residues, with R218Q abolishing binding in the range of the assay altogether. R189 is a particularly notable residue, since the R189Q mutation completely abolishes channel activity of the purified protein, but only slightly reduced channel binding to PI(4, 5)P_2_ with no effect on binding of other PIPs. This residue may thus serve as a lynch-pin, coupling PI(4, 5)P_2_ binding to a transduction mechanism that leads to channel activation. Docking simulations to Kir2.1 models containing R80Q, R82Q, K182Q, K185Q, K187Q, or K188Q mutations qualitatively confirmed the above experimental findings, since none of these single mutations in the binding pocket abolished PI(4, 5)P_2_ binding, however, the K185Q mutation did exhibit the greatest reduction in PIP binding.

Another intriguing question persists: why are some Kir channels (such as Kir2.1) are selectively activated by PI(4, 5)P_2_, while some members (such as Kir3.1/3.4) are less stringent in their PIP sensitivity, and others still (such as Kir6.2) are indiscriminately activated by all PIPs? Unfortunately, no crystal structures of a Kir channel bound to a PIP other than PI(4, 5)P_2_ is available to date. However, some details can be inferred from other experimental data.

Both biochemical and computational approaches indicate that PI(4, 5)P_2_ specific activation arises not from a uniquely low free binding energy for this ligand. Instead, each PIP ligand can bind with in the same overall location with varying energies, but in non-identical conformations (D'Avanzo et al., 2013). Biochemical assays suggests that the key interactions which govern binding of each particular PIP are different. For example, K185, K187, K188, R189, R218, R219, and R312 all markedly affect the binding Kir2.1 channels to various PIPs. However, binding of each PIP isoform appears to be regulated by a different subset of these residues. For example, PI(3, 4, 5)P_3_ binding to Kir2.1 channels was disrupted by K185Q, K187Q, K188Q, K219Q, and R312Q mutations, while PI(5)P and PI(3, 4)P_2_ binding was only disrupted by the R218Q mutation. Ligand docking approaches to identify and compare putative binding sites in homology models of human Kir2.1 channels based on the PI(4, 5)P_2_-bound structure of chicken Kir2.2 channels (PDB: 3SPI), reveal that all PIPs bind within the same general pocket, but with different conformational orientations and rotational freedom. Thus, while they bind in the same pocket, other PIPs do not appear capable of interacting with the same subset of residues as PI(4, 5)P_2_ and thus may not be able to trigger the necessary conformational changes needed to activate Kir2.1 channels. Mono-phosphorylated PIPs appear to interact with lower affinity, but greater conformational freedom, which may lead to entropically driven competitive inhibition. PI(3, 4, 5)P_3_ which binds the site with similar energy as PI(4, 5)P_2_, does sample this conformation occasionally, which may explain why it can activate Kir2.1 channels with about 10% efficacy (Rohacs et al., 1999, 2003; D'Avanzo et al., 2010a). Thus, two mechanisms can conceivably contribute to the different PIP sensitivities in the various Kir channel isoforms: (i) small changes in the channels' sequences may permit the PIP ligands to more frequently sample PI(4, 5)P_2_-like poses, enabling the appropriate interactions with all the residues that would trigger downstream activation of the channel; or (ii) changes in the channels' sequences enable PIP ligands in different orientations to trigger conformational changes that lead to channel activation. Distinguishing between these two mechanisms will require future experimentation. In this direction, attempts to convert the highly PI(4, 5)P_2_ selective channel Kir2.1 to a non-selective Kir6.2 channel phenotype (Rohacs et al., 2003) has thus far met with limited success, through has provided some useful insights. Mutations of the Kir2.1 C-terminal residues to their Kir6.2 equivalent (namely M180F and K185Q) rendered the channels more responsive to activation by PI(3, 4, 5)P_3_ but not PI(3, 4)P_2_, while N216D, L222I, L232V Y242F, and T268I did not affect PIP sensitivity. Interestingly, mutations of N-terminal residues had a greater effect, with D51K, F58H, C76L, and I79L rendering Kir2.1 channels more responsive to activation by PI(3, 4, 5)P_3_ but again not PI(3, 4)P_2_. Notably, these residues are distant from the PIP_2_ binding site seen in the crystal structures. However, it was only when these mutations were combined (D51K-C76L-K185Q; D51K-I179L-K185Q, or D51K-C76L-I79L-K185Q) did Kir2.1 channels become equally sensitive to PI(3, 4, 5)P_3_ as PI(4, 5)P_2_, and somewhat (but still less than 20%) responsive to PI(3, 4)P_2_.

## Structural rearrangements underlying PIP-dependant gating in kir channels

**Figure 2:**
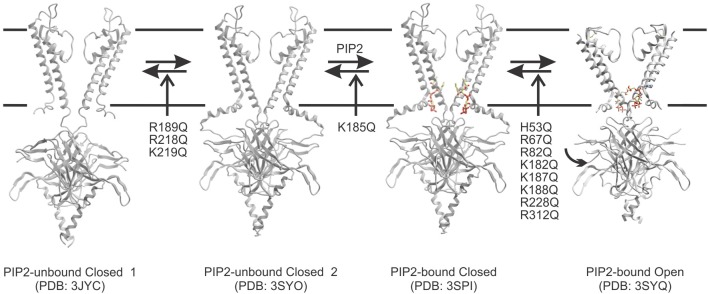
**A proposed model for the predominant pathway of PIP_2_-dependent channel activation**. Kir channels may undergo a conformational change whereby the cytoplasmic domain moves from an extended 3JYC-type closed conformation toward the plasma membrane and interacts through a hydrogen bond network with the slide helix. This leads to a compact PI(4,5)P_2_ unbound structure (similar to what was observed for the Kir3.2 apo structure PDB: 3SYO). Kir2.1 mutations in R189, R218, and K219 appears to disrupt this equilibrium thereby leading to reduced binding of PIP ligands. However, once the transition occurs, this state enables PI(4,5)P_2_ to bind by generating a three-dimensional pocket that can co-ordinate the ligand (PDB: 3SPI). Ligand binding within the pocket appears to be disrupted only by a K185Q mutation in Kir2.1 channels. PI(4,5)P_2_ binding in a particular conformation may then trigger rotation of the S6, and conformational changes in the cytoplasmic domain that lead to channel opening (PDB: 3SYQ). Most mutants that affect PI(4,5)P_2_ sensitivity seem to alter activity through this final transition step.

In the absence of PIP_2_, the cytoplasmic domain of Kir2.2 channels was found to be displaced greater than 10 Å from the slide-helix (PDB: 3JYC) (Tao et al., 2009) (Figure 2) which presumably correlates to the location of the lipid bilayer. The short linker between the TM2 and cytoplasmic domain appeared disordered and elongated. In this conformation, the residues that are key to PIP_2_ binding are too far apart from one another to co-ordinate the lipid ligand. A follow-up structure of Kir2.2 channels bound to 4 PIP_2_ molecules (PDB: 3SPI) indicated that flexible linker becomes structured into a short α-helix and the cytoplasmic domain moves 6 Å upward toward the transmembrane domain, enabling interactions of this domain with the slide helices (Hansen et al., 2011). It had been previously suggested that PIP_2_ may interact with the cytoplasmic domain while it is displaced from the membrane by nearly fully extending out of the bilayer (Haider et al., 2007b; Stansfeld et al., 2009). The implication of this was that the energy of activation was provided by PIP_2_ recoiling to the membrane (Fan and Makielski, 1997; Haider et al., 2007b). However, recent crystal structures of Kir3.2 indicate these channels are capable of adopting a compacted conformation (with the α-helical linker and cytoplasmic domain engaged with the slide helices) even when not complexed with PIP_2_ (Whorton and MacKinnon, 2013) (Figure 2). Furthermore, docking simulations to models of Kir2.1 channels generated using the extended 3JYC structure as a template indicated binding energies on average increased by 3.3 kcal/mol over simulations done using models generated from the compacted 3SPI structure (D'Avanzo et al., 2013). Thus, it is likely that PIP_2_ primarily binds once the channels sample this compacted conformation. Once PIP_2_ binds into this pocket, the appropriate co-ordination between the ligand and side-chains can then trigger the conformational changes that trigger channel activation.

**Figure 3:**
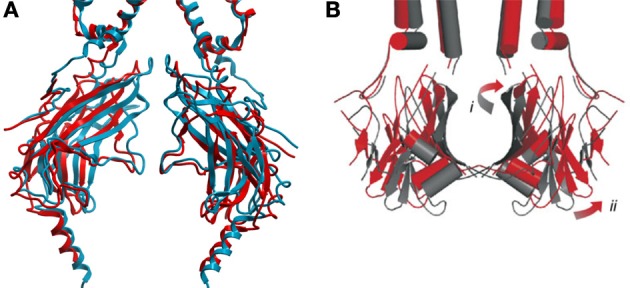
**(A)** Comparison of GIRK2 in the closed PIP_2_ bound conformation (PDB: 3SYA, blue) and in the maximally open (R201A) + PIP_2_ bound conformation (PDB: 3SYQ, red). **(B)** Comparison of the KirBac1.1 channel cytoplasmic domain (subunits A and C) in the closed (PDB: 1P7B, gray) and predicted “open” (red) conformations. Opening requires outward twisting and tilting of the major βI sheet (i), and outward motion of the minor βII sheet (ii) [adapted from Wang et al. (2012)]. Qualitatively, the movements involved in GIRK2 channel opening appear similar to those predicted for the prokaryotic KirBac1.1 channel.

Upon PIP_2_ binding to WT Kir3.2 channels, a small displacement of the protein backbone, where the slide-helix becomes the TM1 or outer helix, could be observed. This was accompanied by a slight rotation of the TM2 or inner helix. In the case of Kir3.2, which also requires the presence of the Gβγ subunit for activation, these changes were not sufficient to fully open the channel gate(s) (Whorton and MacKinnon, 2011). However, in the presence of the Gβγ subunit and PIP_2_, the cytoplasmic domain was observed to be rotated 4° counter-clockwise about the channel axis relative to the transmembrane domains (Whorton and MacKinnon, 2013). This rotation is associated with an unwrapping and splaying of the four TM2 helices to a minimum diameter of 6–7 Å which appears to disorder the four Phe192 residues that forms the narrowest constriction in the closed channel. The addition of an R201A mutation induced a further 4° counter-clockwise rotation in the cytoplasmic domain, and widens the pore diameter at the bottom of the TM2 to 9 Å. The rotation of the entire cytoplasmic domain is also associated to tilting movements of the major βI sheets and rotational movements of the minor βII sheets that make up its structure (Figure 3A).

PIP_2_-dependent closure of bacterial KirBac1.1 channels determined by FRET (Wang et al., 2012) appears qualitatively similar to what can be inferred from the series of eukaryotic crystal structures described above (Figure 3A). In the presence of PIP_2_ residues on the top of the major βI sheet move toward the central pore axis, while residues at the bottom move away. Meanwhile, all residues tested within the minor βII sheet were found to move toward the central axis in the presence of PIP_2_ (Wang et al., 2012). A “cartoon” model of an open KirBac1.1 channel (Figure 3B) developed from the closed structure (PDB: 1P7B) and minimization of the constraints obtained by FRET measurements was generated by tilting of the major βI sheet with the upper end moving away from the pore axis, while the bottom end moved toward it. This induced widening of the upper vestibule. The minor βII sheet is predicted to twist counter-clockwise which resulted in a movement away from the central axis and widening of the cytoplasmic pore.

A recent structure of the KirBac3.1 channel (Bavro et al., 2012) provides further insight into how Kir channels may open. In the open conformation, the TM2 is splayed approximately 20° and rotated along the helical axis approximately 25° compared to the closed conformation (PDB: 2WLJ). This opens the pore diameter at the narrowest constriction (Tyr132) from less than 2 Å to more than 8 Å. The cytoplasmic domain is twisted about the pore axis which displaces the C-linker approximately 5 Å relative to the channels closed conformation, leading to the development of a stabilizing H-bond network between the C-linker, and cytoplasmic domains, specifically the CD-loop and G-loop. This network appears to strengthen the interaction between the cytoplasmic domain and TM domains via the slide helix.

The PIP dependence of KirBac3.1 channels has yet to be determined. Regardless, considering the sequence and structural homology between KirBac1.1 and KirBac3.1 channels (Kuo et al., 2003; Bavro et al., 2012), it is likely similar structural rearrangements occur in these two channels during channel gating. Furthermore, it appears that qualitatively similar movements within the cytoplasmic domain are associated with channel opening in both prokaryotic and eukaryotic Kir channels (Figure 3). Thus, it is still unclear why PIPs can activate eukaryotic Kirs while inhibiting bacterial Kirs. Further experimental approaches are needed to resolve the molecular basis for this intriguing contrary regulatory role of PIPs in prokaryotic and eukaryotic Kir channels, however, we will take this opportunity to speculate on a potential mechanism. It has been suggested that this paradoxical behavior might be the result of missing key residues in the KirBac N-terminal and C-terminal linkers that link the transmembrane and cytoplasmic domains together (D'Avanzo et al., 2010b). Alignments of KirBac and eukaryotic Kir sequences reveal that each of these linkers is longer by 3 residues in the eukaryotic Kirs. Additionally, the C-linker contains two charged residues (K187 and K188 in Kir2.1 channels) which co-ordinate PIP_2_ in the binding pocket and when mutated cause a loss of PIP_2_ activation (Shyng et al., 2000; Lopes et al., 2002; D'Avanzo et al., 2013) though not a loss in PIP_2_ binding. These 3 residue insertions seem to displace the cytoplasmic domain away from the slide helix, an interaction which has been suggested to play a key role in channel gating in both prokaryotic and eukaryotic Kir channels. Mutations which disrupt this interaction can destabilize the open state and favor channel closure, although the ability of these proteins to bind PIP_2_ remains intact (Decher et al., 2007). Thus, the shorter linkers in KirBacs may energetically favor interactions between the slide helix and the cytoplasmic domain, leading to opening of the channel in the absence of PIP_2_. Binding of PIP_2_ to KirBacs may act to destabilize this interaction, through disruption of the hydrogen bond network seen in the C-linker region of the open KirBac3.1 structure (Bavro et al., 2012), thereby separating the cytoplasmic domain from the slide helix, leading to channel closure. Other PIPs may be able to equivalently disrupt these interactions with increased success as the degree of phosphorylation (and thus charge) increases in the headgroup. On the other hand, the longer linker of eukaryotic Kirs would minimize the interaction between the slide helix and the cytoplasmic domain, and thereby keep the channel closed in membranes that lack PIP_2_. Once the eukaryotic channels sample the compact conformation, the presence of PIP_2_ appears to begin nudging the TM2 domain toward opening, and further stabilize the cytoplasmic domain and slide helix interactions necessary for channel opening. The interactions needed for this is dependent on the specific nature of the channel and ligand isoforms.

### Conflict of interest statement

The authors declare that the research was conducted in the absence of any commercial or financial relationships that could be construed as a potential conflict of interest.
